# The Impact of Conservative Versus Pharmacological Management on Maternal and Neonatal Outcomes in Gestational Diabetes: A Systematic Review and Meta-Analysis

**DOI:** 10.7759/cureus.94610

**Published:** 2025-10-15

**Authors:** Eberechukwu Ikwuanusi, Itihad Yafai, Emmanuel Emovon

**Affiliations:** 1 Medicine, University Hospitals Birmingham, Birmingham, GBR; 2 Hospital-Based Medicine, University Hospitals Birmingham, Birmingham, GBR; 3 Obstetrics and Gynaecology, Sandwell and West Birmingham Hospitals, Birmingham, GBR

**Keywords:** gestational diabetes mellitus (gdm), maternal clinical outcomes, neonatal clinical outcomes, pharmacological management, conservative management

## Abstract

Gestational diabetes mellitus (GDM) is a common complication in pregnancy. Its early identification through targeted screening and timely management with lifestyle or pharmacological interventions is essential to optimise outcomes. Current evidence on GDM management is limited, with few studies directly comparing conservative measures with pharmacological therapy. This review evaluates the impact of conservative versus pharmacological management on maternal and neonatal outcomes. Databases and grey literature were searched, and identified studies were screened using pre-defined eligibility criteria. Data were extracted from the included studies, and study quality was appraised using the Critical Appraisal Skills Programme (CASP) tool. Maternal and neonatal outcomes were pooled in a meta-analysis. Conservative management was associated with a 30% reduction in adverse maternal outcomes (risk ratio (RR) 0.70, 95% confidence interval (CI): 0.50-0.98, p=0.04) and a 26% reduction in adverse neonatal outcomes (RR: 0.74, 95% CI: 0.60-0.96, p=0.02). Subgroup analyses revealed no significant differences between pharmacological therapies. Overall, conservative management of GDM appears to improve both maternal and neonatal outcomes compared to pharmacological management. These findings support lifestyle-based interventions as a safe and effective option, though further research is needed to confirm clinical benefits and refine clinical guidance.

## Introduction and background

Gestational diabetes mellitus (GDM) is a well-recognised complication of pregnancy, affecting up to 25% of pregnancies worldwide. Its reported prevalence varies due to differences in ethnicity, maternal age, and the screening and diagnostic methods used [1]. GDM is defined as impaired glucose tolerance resulting in dysregulated glucose levels during pregnancy. It typically resolves postpartum and is diagnosed using lower blood glucose thresholds than those applied outside of pregnancy. However, diagnostic criteria are not uniform globally, contributing to underdiagnosis in some settings [2].

Pregnancy is characterised by substantial physiological adaptations to meet the increasing metabolic demands of the growing fetus. A key change is insulin sensitivity, which fluctuates throughout gestation. In early pregnancy, insulin sensitivity rises to facilitate maternal energy storage, whereas in later pregnancy, placental hormones promote insulin resistance and stimulate β-cell proliferation to maintain glucose homeostasis. In patients with GDM, however, β-cell dysfunction results in both insulin resistance and maternal dysglycaemia [3]. In normal pregnancies, blood glucose levels are slightly elevated and readily cross the placental wall to promote fetal growth and development. This mild insulin resistance then reverts to insulin sensitivity (pre-pregnancy state) a few days after delivery; however, these processes do not always occur adequately, leading to the development of GDM in some pregnancies [4].

In the UK, the National Institute for Health and Care Excellence (NICE) currently recommends targeted screening for GDM in women with known risk factors. Screening with the oral glucose tolerance test (OGTT) is routinely performed at 24-28 weeks, but is usually undertaken earlier in women with a history of GDM, sometimes as early as 16 weeks [4,5]. NICE has established clear guidelines for the diagnosis of GDM, including a fasting plasma glucose level of ≥5.6 mmol/L or a two-hour plasma glucose level of ≥7.8 mmol/L. On diagnosis, women are offered review in a joint diabetic and antenatal clinic for further support and guidance throughout their pregnancy [6].

Following diagnosis, treatment options are explored with the mother. Management can include conservative measures, such as dietary modifications, exercise, and lifestyle changes, or pharmacological therapies, including metformin, insulin, and other medications. Patient education on self-monitoring of blood glucose, along with ongoing management and regular follow-up throughout pregnancy, is essential to ensure optimal diabetes control and reduce the risk of adverse outcomes [7,8]. Maternal complications include pre-eclampsia, preterm birth, higher rates of caesarean section, and infections. Neonatal risks include acute respiratory distress syndrome (ARDS), small for gestational age (SGA), and macrosomia [9]. The wide range of significant maternal and neonatal complications underscores the importance of effective GDM management, guided by the best available evidence.

Current evidence remains limited, with many studies focusing either on lifestyle interventions or on comparisons between different pharmacological agents, rather than directly evaluating conservative measures, such as diet and exercise, against pharmacotherapy [10,11]. This review, therefore, aims to compare the impact of conservative versus pharmacological management on maternal and neonatal outcomes in GDM, to guide evidence-based clinical decision-making.

## Review

Methodology 

Study Design

This project adhered to the Preferred Reporting Items for Systematic Reviews and Meta-Analyses (PRISMA) guidelines [12] and was registered with the International Prospective Register of Systematic Reviews (PROSPERO CRD420251142634). The PICOS (Patient/Problem, Intervention, Comparison, and Outcome, with Study Design) framework was used to develop the research questions, search terms, and eligibility criteria applied in this study.

Search Strategy

The following databases and grey literature sources were searched: MEDLINE (Ovid), PubMed, Cochrane Library, and Ethos. Searches were conducted on July 26, 2025, using the following predetermined search terms and their variations: “gestational diabetes” AND “conservative treatment” AND “pharmacotherapy” AND (“maternal outcomes” OR “neonatal outcomes”) (Appendix 1). Reference lists of included studies were also reviewed to identify additional relevant publications.

Eligibility Criteria

Rayyan citation software was used to manage studies retrieved from databases, grey literature, and reference lists [13]. Duplicates were removed, and titles and abstracts were independently screened by two researchers using the selection criteria (Table 1). Full texts were then independently screened against the same criteria, with any disagreements resolved by an independent reviewer.

**Table 1:** Selection criteria The inclusion and exclusion criteria were applied during the screening of titles and abstracts, and then to full study screening GDM: gestational diabetes mellitus

Inclusion criteria	Exclusion criteria
Pregnant women diagnosed with GDM	Individuals who are not pregnant or do not have a diagnosis of GDM
Infants born to mothers diagnosed with GDM	Infants not born to mothers diagnosed with GDM
Studies comparing conservative management (e.g., diet, exercise) with pharmacological management for GDM	Studies where conservative management is not compared to pharmacological management
Studies reporting the prevalence of maternal and neonatal clinical outcomes (e.g., pre-eclampsia, macrosomia)	Studies not reporting the prevalence of maternal or neonatal outcomes
Primary comparative studies	Non-primary studies or studies without a comparative component
Human studies	Non-human studies
Studies published in English	Studies not published in English

Data Extraction and Quality Appraisal

Data from each included study were extracted using a standardised form to ensure accuracy and consistency. Two independent reviewers collected information on study characteristics, participant demographics, type of intervention, and key maternal and neonatal outcomes (e.g. pre-eclampsia, macrosomia). Any discrepancies were resolved by consultation with an independent reviewer. The quality of the included studies was assessed using the Critical Appraisal Skills Programme (CASP) tool [14,15]. Methodological quality was independently appraised by two reviewers.

Data Analysis

Data extracted from the included studies were combined in a meta-analysis using Review Manager 5.4 [16]. The effects of conservative management compared to pharmacological interventions on maternal and neonatal outcomes were expressed as risk ratios (RR) with 95% confidence intervals (CI). To preserve the integrity of randomisation and provide unbiased comparisons, an intention-to-treat analysis was conducted. Heterogeneity between studies was assessed using the I² statistic. For studies including more than one intervention group, the conservative group was appropriately divided to allow comparisons with each treatment group without counting the control group more than once, in accordance with Cochrane Handbook recommendations [17].

Results

Search Results

**Figure 1:**
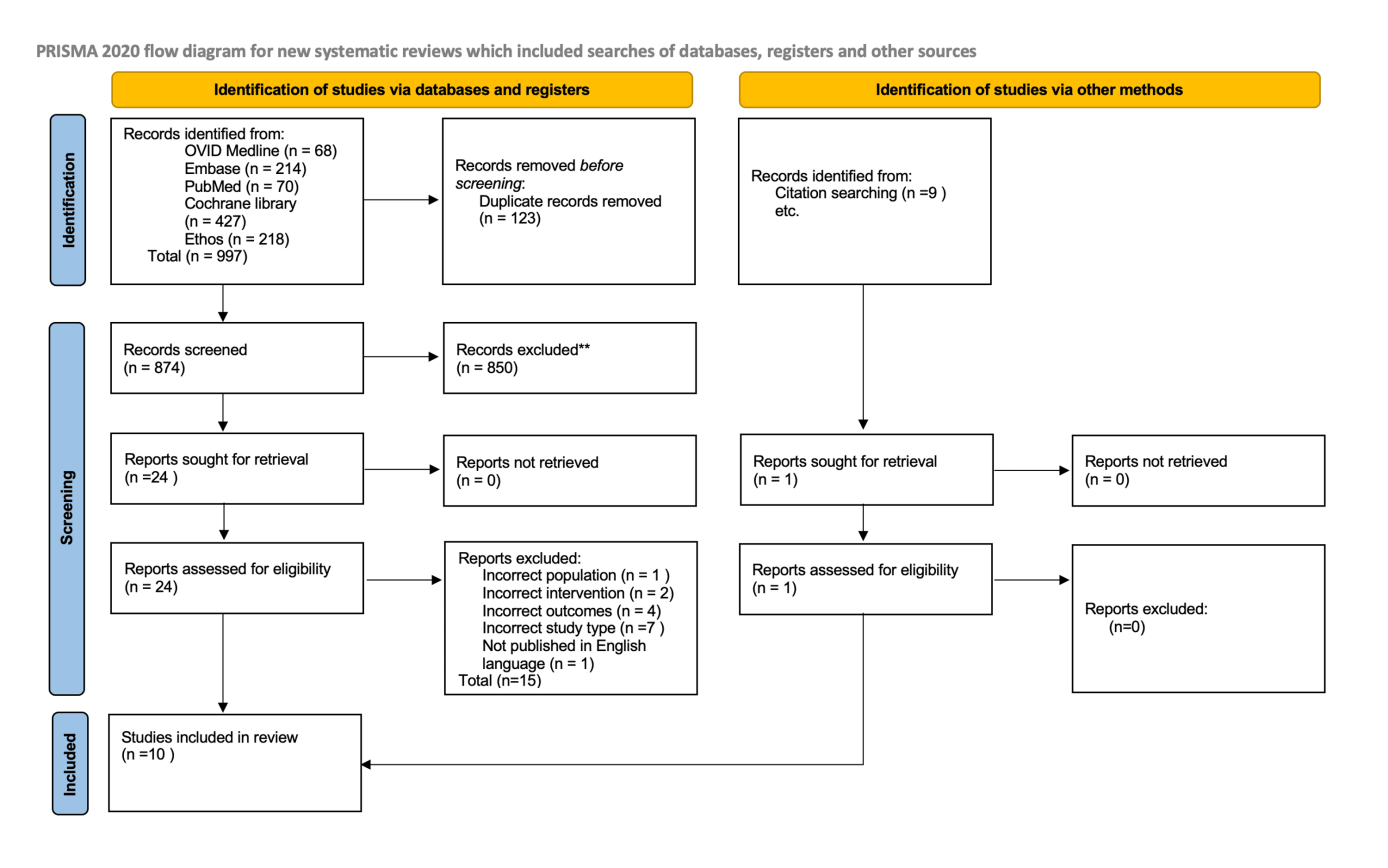
PRISMA flow diagram depicting the study selection process PRISMA: Preferred Reporting Items for Systematic Reviews and Meta-Analyses

A total of 997 studies were identified through literature searching and screening against eligibility criteria. Reasons for study exclusion are provided in Appendix 2. A total of 10 studies were included in this review (Figure 1), and an overview of their characteristics is presented in Table 2 [18-27]. The 10 included studies comprised a mix of randomised controlled trials (RCTs) and observational cohorts evaluating dietary, insulin, and oral therapies for GDM, with sample sizes ranging from fewer than 100 to 1,300 women. Overall, 3,044 women with GDM were included, representing nine countries: the United Kingdom, United States of America, France, Qatar, Italy, Sweden, Macedonia, and Malaysia. Pharmacological interventions included insulin, metformin, glibenclamide, and glyburide. Maternal outcomes focused on mode of delivery, induction of labour, and gestational age at birth, while neonatal outcomes included birthweight, large for gestational age (LGA)/macrosomia, hypoglycaemia, neonatal ICU (NICU) admission, and respiratory complications.

**Table 2:** Summary of study characteristics and outcomes* ^*^[18-27] Outcome code system: Maternal outcomes (M): M1: glycaemic control (HbA1c); M2: need for additional therapy/escalation (switch to insulin); M3a: SVD; M3b: IOL; M3c: elective/planned cesarean section (C-section); M3d: emergency/non-scheduled C-section; M4: hypertensive disorders; M5: maternal weight gain: M6: adverse drug effects; M7: other obstetric complications (e.g. polyhydramnios, preterm labour trigger etc.) Neonatal outcomes (N): N1: birthweight/macrosomia (>4 kg or >90th centile); N2: LGA; N3: SGA; N4: preterm birth (<37 weeks); N5: neonatal hypoglycaemia; N6: NICU admission; N7: respiratory distress/pulmonary maladaptation; N8: shoulder dystocia/birth trauma; N9: perinatal mortality/stillbirth; N10: neonatal jaundice; N11: neonatal compromise (e.g., low Apgar, foetal distress); N12: anthropometry/body composition RCT: randomised controlled trial; GDM: gestational diabetes mellitus; SVD: spontaneous vaginal delivery; IOL: induction of labour; LGA: large for gestational age; SGA: small for gestational age; NICU: neonatal intensive care unit

Study	Design	Population	Intervention/exposure	Comparator	Outcomes
Blachier et al., 2014 [18]	Prospective cohort study	1351 women with GDM (n=253 insulin; n=1108 diet)	Insulin	Diet/lifestyle advice	Maternal: M3a, M3d. Neonatal: N5, N8, N1
Casey et al., 2015 [19]	RCT	375 women with GDM (n=189 glyburide; n=186 placebo)	Glyburide	Placebo and diet/lifestyle advice	Maternal: M3a, M3b. Neonatal: N2, N3 N6, N5, N8, N1
Coustan and Lewis 1978 [20]	RCT	72 women with GDM (n=34 control; n=27 insulin; n=11 diet)	Insulin	Diet/control	Maternal: M3. Neonatal: N11, Apgar <7 (1 or 5 minutes)
D’Souza et al., 2025 [21]	Retrospective cohort study	649 women with GDM (n=211 metformin; n=438 diet)	Metformin	Diet	Maternal: M3b, M3c, M3d, M7, M4. Neonatal: N2, N6, N5, N7
Holt et al., 2008 [22]	Observational study	144 women with GDM (n=44 glibenclamide; n=45 insulin; n=55 diet)	Glibenclamide/insulin	Diet	Maternal: M3a, M3b. Neonatal: N5, N10, N6, Apgar at 1 or 5 minutes
Mello et al., 1997 [23]	Observational cohort study	217 women with GDM (n=121 insulin and diet; n=96 diet alone)	Insulin and diet	Diet	Maternal: M3a. Neonatal: N2, N3, anthropometry
Persson et al., 1985 [24]	RCT	202 women with GDM (n=97 insulin and diet; n=105 diet alone)	Insulin and diet	Diet	Neonatal: N7, N5, N2
Simeonov-Krestevska et al., 2018 [25]	Observational cohort study	349 women with GDM (n=48 metformin; n=101 insulin; n=200 diet)	Metformin/insulin	Diet	Maternal: M3a, M3c, M3d, M7. Neonatal: N2, N3, N5, Apgar at 5 minutes
Tew et al., 2022 [26]	Double-blind placebo-controlled RCT	106 women with GDM (n=53 metformin; n=53 diet/lifestyle advice)	Metformin	Diet/lifestyle advice	Maternal: M7, M1. Neonatal: N1
Weiss et al., 1988 [27]	Observational cohort study	423 women with GDM (n=253 insulin and diet; n=170 diet alone)	Insulin and diet	Diet	Neonatal: N5, N7, N4, N1

Maternal Outcomes

**Figure 2:**
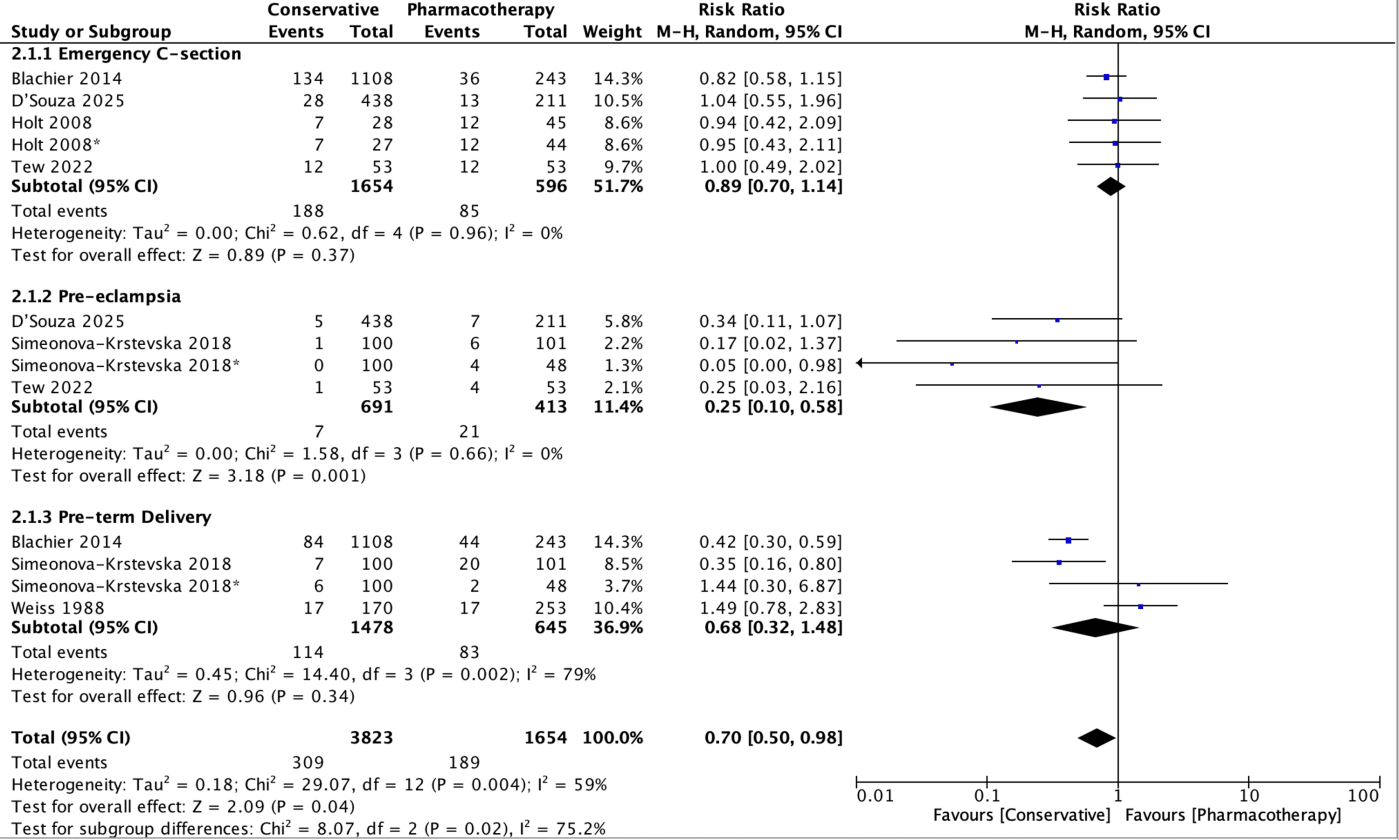
Forest plot of maternal outcomes RR of adverse maternal outcomes in conservative versus pharmacotherapy groups (RR: 0.70, 95% CI: 0.50–0.98, p=0.04). There is statistically significant heterogeneity between the included studies (I²=59%, p=0.004) [18,21,22,25-27] RR: risk ratio; CI: confidence interval

**Figure 3:**
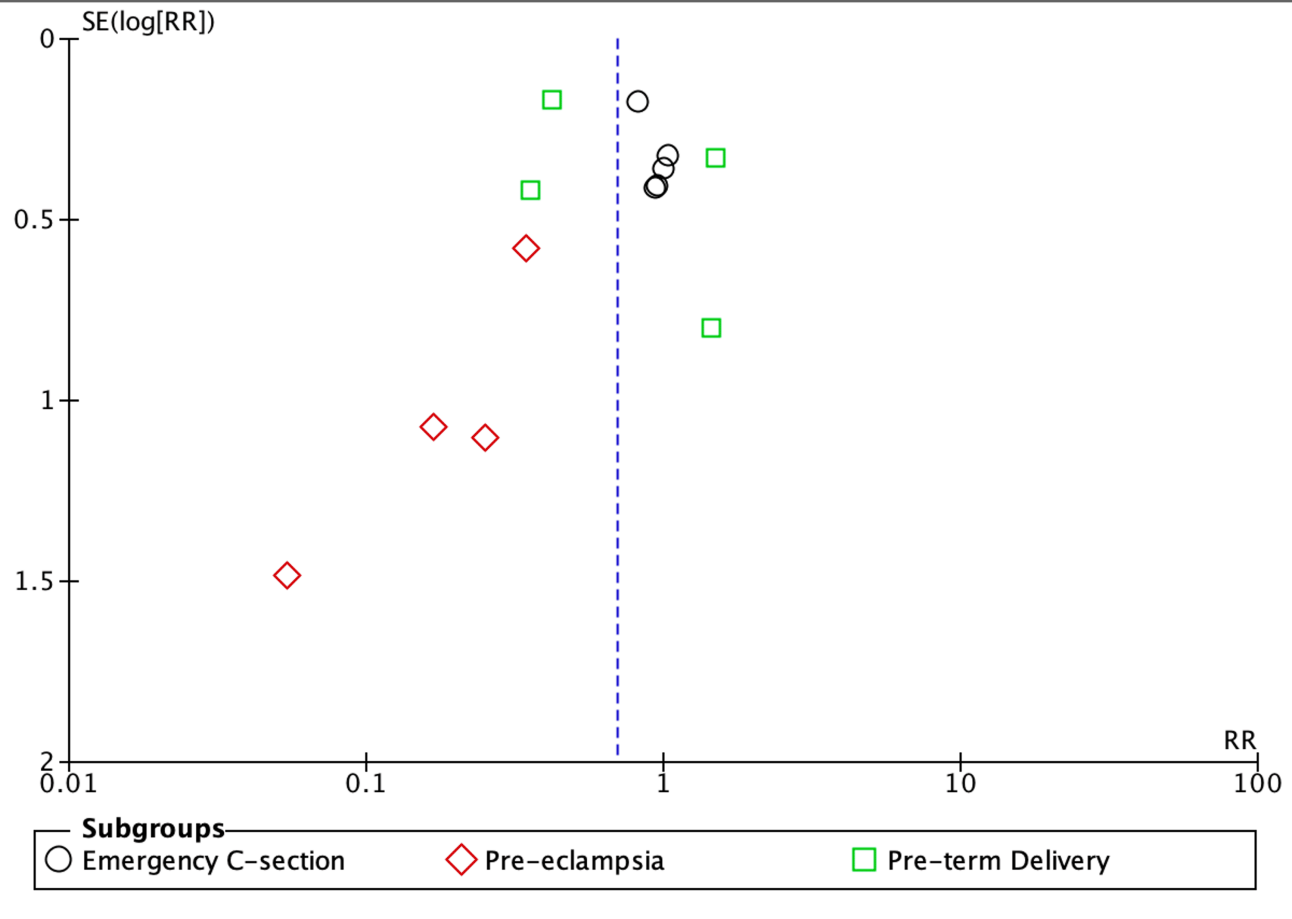
Funnel plot of maternal outcomes Funnel plot illustrating an asymmetrical distribution of studies, suggestive of potential publication bias [18,21,22,25-27]

An overview of maternal outcomes is shown in Figures 2, 3.

Neonatal Outcomes

**Figure 4:**
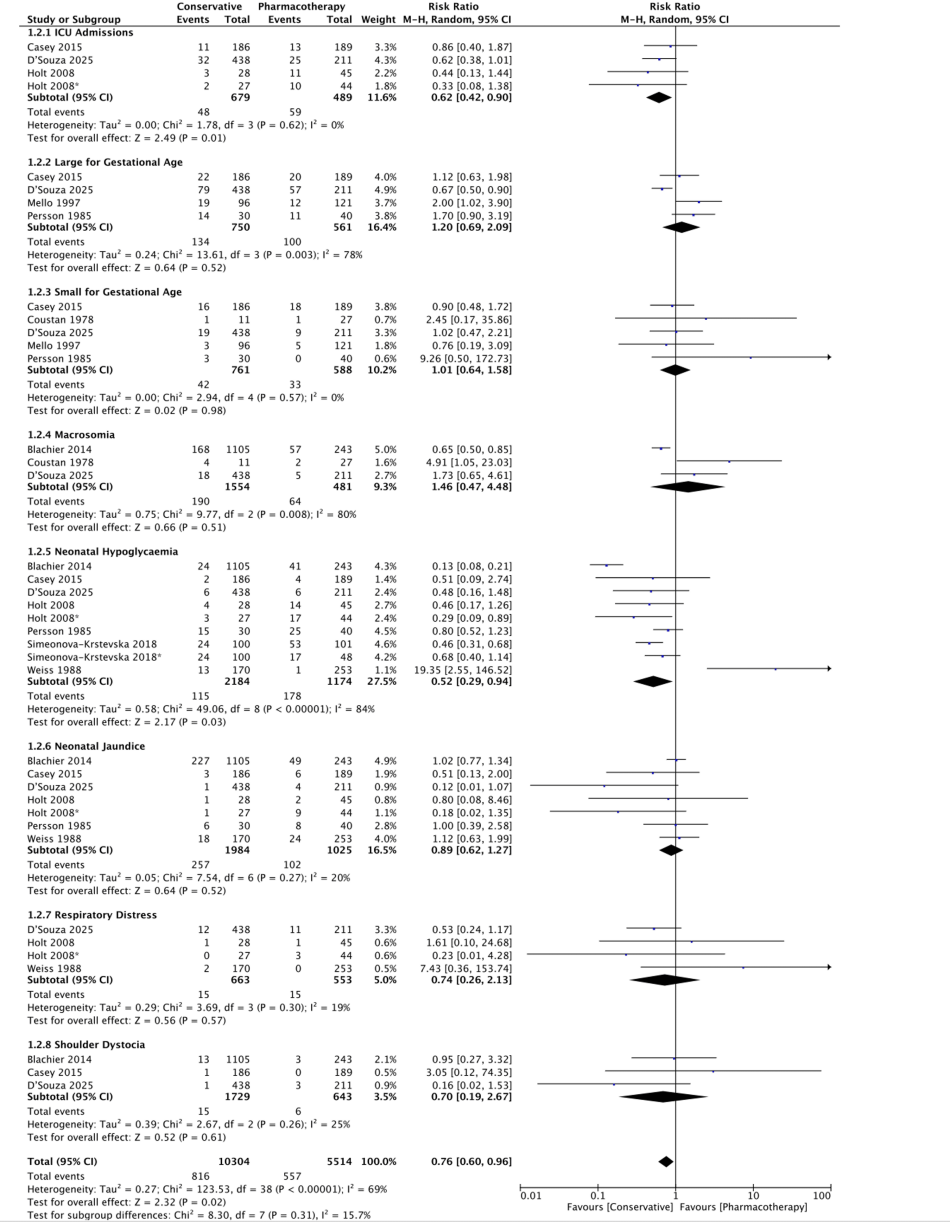
Forest plot of neonatal outcomes RR of adverse neonatal outcomes in conservative versus pharmacotherapy groups (RR: 0.74, 95% CI: 0.60–0.96, p=0.02). There is statistically significant heterogeneity between the included studies (I²=69%, p<0.0001) [18-25,27] RR: risk ratio; CI: confidence interval

**Figure 5:**
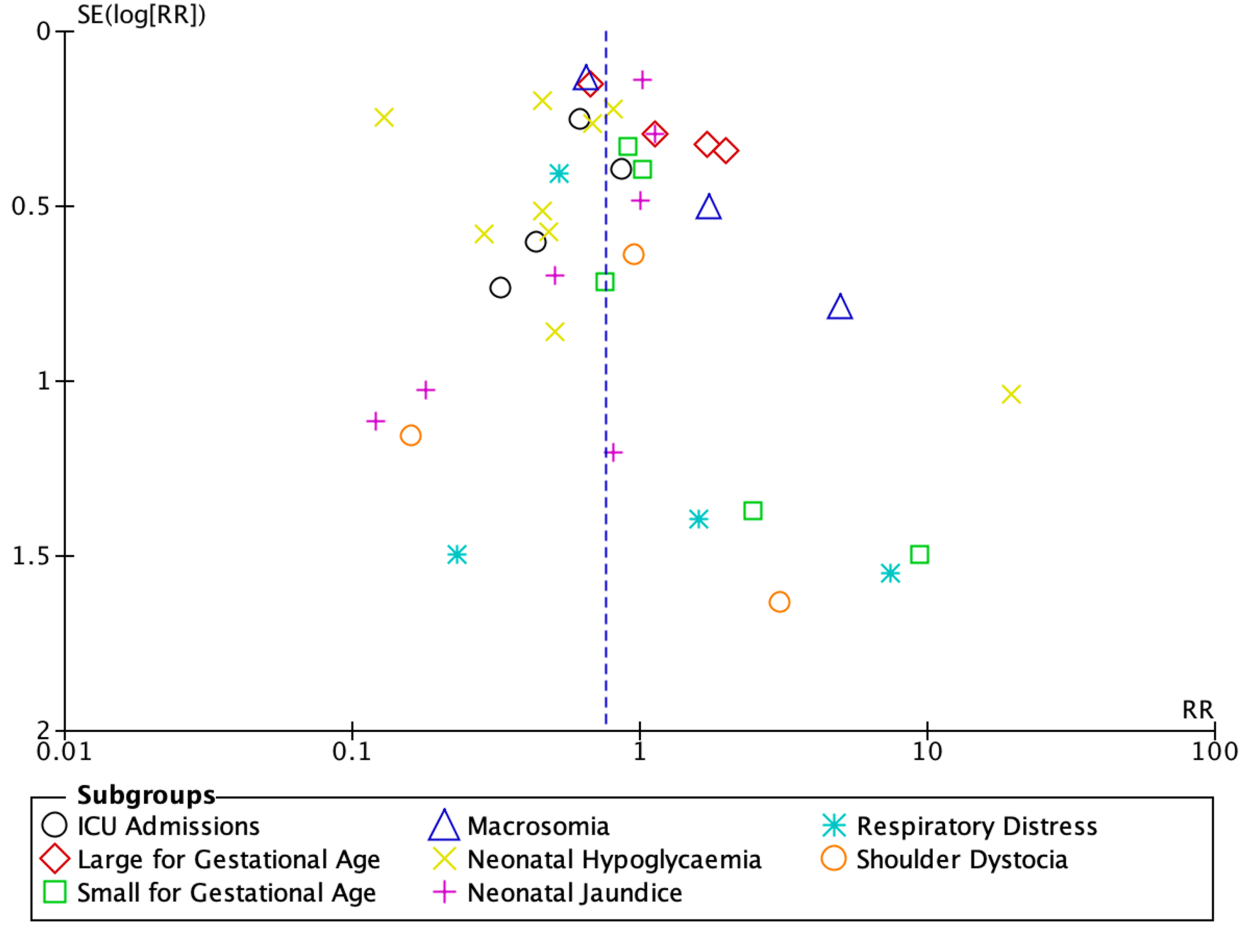
Funnel plot of neonatal outcomes Funnel plot showing a relatively symmetrical distribution of studies, suggesting minimal publication bias for neonatal outcomes [18-25,27]

An overview of neonatal outcomes is shown in Figures 4, 5.

Quality Appraisal

Across the included studies, several methodological strengths were highlighted using the CASP checklist. Most studies addressed clinically relevant research questions with clearly defined interventions and outcomes. Objective outcome data were obtained from hospital or clinical records, reducing the risk of measurement error and recall bias. Randomisation was employed in several trials, such as Persson et al. and Casey et al., helping to minimise selection bias and strengthen validity. Many studies also adjusted for key baseline characteristics, including ethnicity, BMI, and other demographic factors, improving comparability between groups. Where randomisation was not ethically or logistically feasible, observational study designs were used. Although inherently more prone to bias, these designs allowed evaluation of interventions in real-world clinical settings, increasing external validity and generalisability to the broader gestational diabetes population.

Despite these strengths, several common limitations were identified. Not all studies were randomised, and in many observational cohorts, treatment allocation was based on disease severity. Women with more severe or poorly controlled GDM were more likely to receive pharmacological therapy, while those with milder disease were managed with diet alone. This introduces confounding by indication, making it difficult to determine whether observed outcomes were due to the intervention or baseline differences in disease severity. Adherence posed additional challenges, as patient compliance with dietary management was difficult to measure, and the quality or accessibility of dietary advice may have varied. Similarly, pharmacological arms often involved variations in dosing regimens and adherence that were not fully captured, potentially influencing outcomes. Other limitations included relatively small sample sizes, which reduced statistical power, and the predominance of non-diverse populations, limiting the generalisability of findings to broader, more heterogeneous groups.

Discussion

Maternal and Neonatal Outcomes

Overall, conservative management was associated with a 30% reduction in the risk of adverse maternal outcomes compared to pharmacological management, which was statistically significant (RR: 0.70, 95% CI: 0.50-0.98, p=0.04) (Figure 2). Notably, the risk of pre-eclampsia was reduced by 75% with conservative management (RR: 0.25, 95% CI: 0.10-0.58, p=0.001). A study by Teede et al. supports this, showing that lifestyle interventions, including diet and physical activity, were associated with a 19% reduction in adverse maternal outcomes (OR: 0.81, 95% CI: 0.69-0.95) [28].

Regarding neonatal outcomes, conservative management also appeared more effective. There was a 26% reduction in the risk of adverse neonatal outcomes in the conservative group compared to the pharmacotherapy group (RR: 0.74, 95% CI: 0.60-0.96, p=0.02) (Figure 4). Statistically significant reductions were also observed in the risk of ICU admission (38% reduction; RR: 0.62, 95% CI: 0.43-0.90, p=0.01) and neonatal hypoglycaemia (48% reduction; RR: 0.52, 95% CI: 0.29-0.94, p=0.03). A study by Wang et al. supports these findings, demonstrating that prenatal lifestyle interventions, including dietary modifications, significantly reduced the risk of neonatal hypoglycaemia (RR: 0.73, 95% CI: 0.54-0.98) [29].

Subgroup analyses based on different pharmacological therapies revealed no statistically significant differences in outcomes, as indicated by overlapping confidence intervals and p-values greater than 0.05 (Appendix 3). This suggests that the choice of pharmacological agent may not substantially affect maternal or neonatal outcomes.

Overall, the findings highlight the potential advantages of conservative management in improving both maternal and neonatal outcomes, with significant reductions in pre-eclampsia, neonatal ICU admissions, and neonatal hypoglycaemia rates. These findings underscore the clinical significance of conservative management and reinforce the importance of prioritising lifestyle-based strategies before escalating to pharmacological therapy.

Quality Appraisal of Systematic Review

This systematic review has several strengths. A comprehensive search of multiple databases, including grey literature, was conducted to reduce publication bias and capture a wide range of studies. Reference lists of included articles were also screened to identify additional eligible studies. Two independent reviewers conducted study selection, data extraction, and quality assessment, enhancing reliability and minimising bias. A meta-analysis was performed to provide pooled estimates, offering more precise insights into maternal and neonatal outcomes. Many studies demonstrated moderate-to-high validity according to the CASP checklist, strengthening the overall quality of evidence.

However, limitations exist. Variability in GDM definitions and diagnostic criteria, inclusion of diet within intervention groups, and the preferential use of pharmacological therapy for more severe cases introduced potential confounding factors. Ethical constraints prevent randomising women needing pharmacotherapy to conservative management alone. Additionally, there was moderate heterogeneity between the included studies, potentially due to differences in patient characteristics, which should be considered when interpreting the pooled results (Figures 2, 4). Evidence of publication bias was observed for maternal outcomes (Figure 3). Finally, because multiple correlated outcomes were reported for the same participants, it was not appropriate to combine all outcomes. Therefore, drug-based subgroup analyses were conducted for selected key maternal and neonatal outcomes (Appendix 3).

Future Research Directions

Future research should address these gaps by including more diverse populations and recruiting women with well-controlled GDM at baseline to enable robust randomisation and reduce confounding by indication. Standardised outcome reporting, consistent adherence measurement, and detailed subgroup analyses are needed to strengthen causal inference. Such studies will enhance the reliability, generalisability, and clinical applicability of findings, supporting informed and personalised management of GDM.

## Conclusions

The findings of this systematic review and meta-analysis suggest that conservative management, particularly lifestyle interventions such as diet and physical activity, offers clinically meaningful benefits for women with GDM and their infants. Conservative management was associated with significant reductions in pre-eclampsia, neonatal ICU admissions, and neonatal hypoglycaemia, supporting its use as a first-line strategy where appropriate. Looking forward, there is a clear need for high-quality trials in diverse populations to enhance generalisability. Future studies should recruit women with well-controlled GDM at baseline and adopt standardised reporting with subgroup analyses to reduce bias and better compare conservative and pharmacological strategies. These insights can help guide personalised, evidence-based management to optimise maternal and neonatal outcomes in GDM.

## Figures and Tables

**Figure 6:**
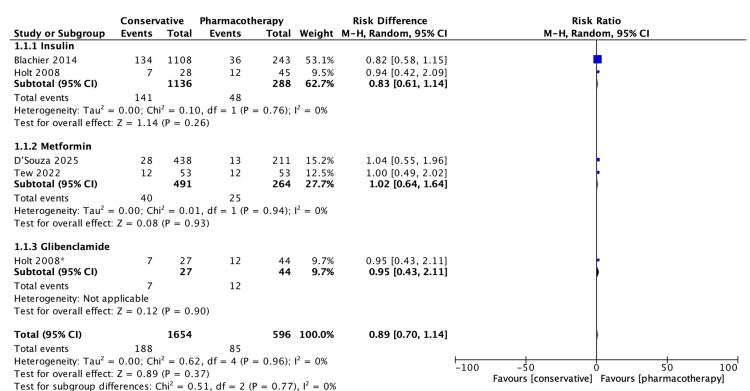
Subgroup analysis of pharmacological management for maternal outcome (emergency C-sections) Clinical outcomes did not differ significantly across the pharmacological management subgroups (p=0.77) [18,21,22,26] CI: confidence interval

**Figure 7:**
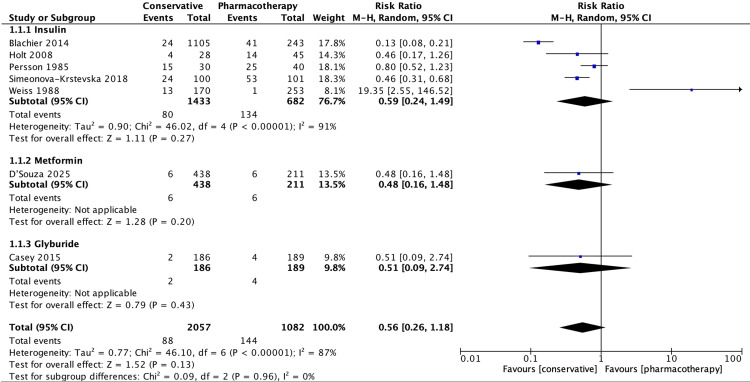
Subgroup analysis of pharmacological management for neonatal outcome (neonatal hypoglycaemia) Clinical outcomes did not differ significantly across the pharmacological management subgroups (p=0.96) [18,19,21,22,24,25,27] CI: confidence interval

## Appendices

1. Search strategy

1.1. Search Terms

**Table 3:** Search terms The search terms and their variations were used for database and grey literature searching

Area	Search term
Gestational diabetes	Gestational diabetes OR GDM OR diabetes in pregnancy OR pregnancy-induced diabetes
Conservative management	Conservative treatment OR conservative management OR diet OR exercise OR lifestyle intervention OR lifestyle modification
Pharmacotherapy	Pharmacotherapy OR insulin OR metformin OR glyburide OR glibenclamide OR hypoglycaemic agent OR drug therapy OR medical therapy
Maternal outcomes	Maternal outcome OR maternal health OR maternal complications OR pregnancy outcome OR pregnancy complication OR weight gain OR hypertensive disorders OR pregnancy-induced hypertension OR pre-eclampsia OR eclampsia OR mode of delivery OR caesarean section OR cesarean section OR labour induction OR preterm labour OR preterm delivery OR preterm birth OR shoulder dystocia OR birth trauma OR maternal mortality OR postpartum haemorrhage OR PPH OR perianal tear OR episiotomy
Neonatal outcomes	Neonatal outcome OR infant outcome OR birth outcome OR macrosomia OR large for gestational age OR LGA OR small for gestational age OR SGA OR intrauterine growth restriction OR IUGR OR low birth weight OR neonatal hypoglycaemia OR hypoglycaemia in newborn OR neonatal hyperbilirubinemia OR neonatal jaundice OR neonatal intensive care OR neonatal mortality OR perinatal mortality OR stillbirth OR neonatal death OR respiratory distress

1.2. Search Strategy

**Table 4:** Search strategy Example search strategy: Ovid MEDLINE(R) ALL <1946 to July 25, 2025>

No.	Search term	Results
1	Gestational diabetes.mp. or Diabetes, Gestational/	28354
2	Limit 1 to abstracts	26124
3	GDM.mp.	14190
4	Limit 3 to abstracts	14128
5	Diabetes in pregnancy.mp.	1733
6	Limit 5 to abstracts	1459
7	Diabetes in pregnancy.mp.	1733
8	Limit 7 to abstracts	1459
9	Diabetes in pregnancy.mp.	1733
10	Limit 9 to abstracts	1459
11	Pregnancy-induced diabetes.mp. or Diabetes, Gestational/	18129
12	Limit 11 to abstracts	16341
13	2 or 4 or 6 or 8 or 10 or 12	27329
14	Conservative treatment.mp. or Conservative Treatment/	43644
15	Limit 14 to abstracts	40121
16	Conservative management.mp. or Conservative Treatment/	26340
17	Limit 16 to abstracts	24594
18	Diet/or diet.mp.	610196
19	Limit 18 to abstracts	528040
20	Exercise/ or exercise.mp.	478898
21	Limit 20 to abstracts	424891
22	Lifestyle intervention.mp.	7297
23	Limit 22 to abstracts	7094
24	Lifestyle modification.mp.	6549
25	Limit 24 to abstracts	6435
26	15 or 17 or 19 or 21 or 23 or 25	975900
27	Pharmacotherapy.mp. or Drug Therapy/	72409
28	Limit 27 to abstracts	49178
29	Insulin.mp. or Insulin/	499414
30	Limit 29 to abstracts	438020
31	Metformin.mp. or Metformin/	34723
32	Limit 31 to abstracts	31711
33	Glyburide.mp. or Glyburide/	7283
34	Limit 33 to abstracts	6463
35	Glibenclamide.mp. or Glyburide/	11421
36	Limit 35 to abstracts	10549
37	Hypoglycaemic agent.mp.	268
38	Limit 37 to abstracts	249
39	Drug therapy.mp. or Drug Therapy/	2923524
40	Limit 39 to abstracts	2216128
41	Medical therapy.mp.	36384
42	Limit 41 to abstracts	34750
43	28 or 30 or 32 or 34 or 36 or 38 or 40 or 42	2643282
44	Maternal outcome.mp.	1655
45	Limit 44 to abstracts	1632
46	Maternal health.mp.	30986
47	Limit 46 to abstracts	22786
48	Maternal complications.mp.	3458
49	Limit 48 to abstracts	3409
50	Weight gain.mp. or Weight Gain/	99069
51	Limit 50 to abstracts	95418
52	Hypertensive disorders.mp.	7358
53	Limit 52 to abstracts	7044
54	Preeclampsia.mp. or Pre-Eclampsia/	52127
55	Limit 54 to abstracts	40139
56	Pregnancy-induced hypertension.mp. or Hypertension, Pregnancy-Induced/	9487
57	Limit 56 to abstracts	8759
58	Eclampsia/ or eclampsia.mp.	49874
59	Limit 58 to abstracts	36089
60	Mode of delivery.mp.	10163
61	Limit 60 to abstracts	9992
62	Pregnancy outcome.mp. or Pregnancy Outcome/	70048
63	Limit 62 to abstracts	62244
64	Caesarean section.mp. or Cesarean Section/	65293
65	Limit 64 to abstracts	47604
66	Labor, Induced/ or labour induction.mp.	10881
67	Limit 66 to abstracts	6561
68	Obstetric Labor, Premature/ or preterm labour.mp.	15167
69	Limit 68 to abstracts	10970
70	Preterm delivery.mp.	13365
71	Limit 70 to abstracts	13061
72	Pregnancy complication.mp. or Pregnancy Complications/	102667
73	Limit 72 to abstracts	61572
74	Preterm birth.mp. or Premature Birth/	38500
75	Limit 74 to abstracts	35902
76	Shoulder dystocia.mp. or Shoulder Dystocia/	1876
77	Limit 76 to abstracts	1637
78	Birth Injuries/ or birth trauma.mp.	6579
79	Limit 78 to abstracts	3643
80	Maternal mortality.mp. or Maternal Mortality/	20599
81	Limit 80 to abstracts	14688
82	Postpartum Hemorrhage/ or postpartum haemorrhage.mp.	10327
83	Limit 82 to abstracts	7261
84	PPH.mp.	6797
85	Limit 84 to abstracts	6708
86	Perianal tear.mp. or Obstetric Labor Complications/	17756
87	Limit 86 to abstracts	8680
88	Episiotomy.mp. or Episiotomy/	4182
89	Limit 88 to abstracts	3258
90	45 or 47 or 49 or 51 or 53 or 55 or 57 or 59 or 61 or 63 or 65 or 67 or 69 or 71 or 73 or 75 or 77 or 79 or 81 or 83 or 85 or 87 or 89	352798
91	Neonatal outcome.mp.	5497
92	Limit 91 to abstracts	5377
93	Infant outcome.mp.	419
94	Limit 93 to abstracts	399
95	Fetal Macrosomia/ or macrosomia.mp.	6743
96	Limit 95 to abstracts	6289
97	Birth Weight/ or large for gestational age.mp.	48749
98	Limit 97 to abstracts	38602
99	LGA.mp.	3830
100	Limit 99 to abstracts	3815
101	Small for gestational age.mp.	17883
102	Limit 101 to abstracts	16804
103	Intrauterine growth restriction.mp. or Fetal Growth Retardation/	23930
104	Limit 103 to abstracts	20968
105	IUGR.mp.	6914
106	Limit 105 to abstracts	6838
107	Low birth weight.mp. or Infant, Low Birth Weight/	51373
108	Limit 107 to abstracts	45000
109	Neonatal hypoglycaemia.mp.	578
110	Limit 109 to abstracts	498
111	Hypoglycaemia in newborn.mp.	15
112	Limit 111 to abstracts	13
113	Neonatal hyperbilirubinemia.mp. or Hyperbilirubinemia, Neonatal/	2343
114	Limit 113 to abstracts	1883
115	Birth outcome.mp.	1476
116	Limit 115 to abstracts	1450
117	Neonatal jaundice.mp. or Jaundice, Neonatal/	7468
118	Limit 117 to abstracts	4109
119	Neonatal intensive care.mp. or Intensive Care, Neonatal/	34080
120	Limit 119 to abstracts	31645
121	Neonatal mortality.mp. or Infant Mortality/	36949
122	Limit 121 to abstracts	22916
123	Perinatal mortality.mp. or Perinatal Mortality/	12180
124	Limit 123 to abstracts	10289
125	Stillbirth.mp. or Stillbirth/	15509
126	Limit 125 to abstracts	13630
127	Neonatal death.mp. or Perinatal Death/	8260
128	limit 127 to abstracts	7352
129	Respiratory Distress Syndrome/ or respiratory distress.mp.	77585
130	limit 129 to abstracts	64633
131	92 or 94 or 96 or 98 or 100 or 102 or 104 or 106 or 108 or 110 or 112 or 114 or 116 or 118 or 120 or 122 or 124 or 126 or 128 or 130	226019
132	90 or 131	514272
133	26 and 43	133522
134	13 and 132 and 133	843
135	limit 134 to (humans and comparative study)	68

2. Reasons for exclusion

**Table 5:** Reasons for exclusions Reasons for excluding studies after full-text screening

Study	Reason for exclusion
Abbassi-Ghanavati M, Casey B, Shivvers S, Tudela C, McIntire D, Leveno K. Randomized trial of glyburide plus diet compared to placebo plus diet in women with gestational diabetes. Am J Obstet Gynecol. 2014;210(1):S179	Incorrect study type
Bung P, Artal R, Khodiguian N. Regular exercise therapy in disorders of carbohydrate metabolism in pregnancy: results of a prospective, randomized longitudinal study. Geburtshilfe Frauenheilkd. 1993;53(3):188-93	Not published in the English language
Dempsey A, Mumby C, Bernatavicius G, Roberts SA, Myers JE. Metformin treatment vs a diabetes model of prenatal care in women with mild fasting hyperglycemia diagnosed in pregnancy: a feasibility study. J Matern Fetal Neonatal Med. 2022;35(23):4469-77	Incorrect intervention
Dodd JM, Louise J, Deussen AR. Metformin and dietary advice for pregnant women who are overweight or obese to promote gestational restriction of weight: the GROW randomized trial. Diabetes. 2018;67(Suppl 1):A379-80	Incorrect population
Dunne F, Newman C, Devane D, Smyth A, Alvarez-Iglesias A, Gillespie P, et al. A randomised placebo-controlled trial of the effectiveness of early metformin in addition to usual care in the reduction of gestational diabetes mellitus effects (EMERGE): study protocol. Trials. 2022;23(1):795	Incorrect study type
Gajardo FM, Candell MB, Moglia MJ, Quezada IS, Alvarez C. Diet versus metformin plus diet for the treatment of mild gestational diabetes in obese patients. Int J Gynaecol Obstet. 2018;143(Suppl 1):565	Incorrect study type
ISRCTN10845466. Metformin in mild gestational diabetes mellitus: a double-blind placebo-controlled randomised trial. Cochrane Central Register of Controlled Trials (CENTRAL). 2019;3	Incorrect study type
Jeelani H, Jabeen F, Mushtaq S. Perinatal morbidity and mortality in gestational diabetes mellitus. JK Pract. 2015;20(3):30-4	Incorrect outcomes
Joy S, Roman A, Istwan N, et al. The effect of maternal obesity on pregnancy outcomes of women with gestational diabetes controlled with diet only, glyburide, or insulin. Am J Perinatol. 2012;29(8):643-648	Incorrect outcomes
Karkkainen H, Laitinen T, Heiskanen N, Saarelainen H, Valtonen P, Lyyra-Laitinen T, et al.: Need for insulin to control gestational diabetes is reflected in the ambulatory arterial stiffness index. BMC Pregnancy Childbirth. 2013;13:9	Incorrect outcomes
Landon MB, Spong CY, Thom E, Carpenter MW, Ramin SM, Casey B, et al. A multicenter, randomized trial of treatment for mild gestational diabetes. N Engl J Med. 2009;361(14):1339-48	Incorrect intervention
Maresh M, Gillmer MD, Beard RW, Alderson CS, Bloxham BA, Elkeles RS. The effect of diet and insulin on metabolic profiles of women with gestational diabetes mellitus. Diabetes. 1985;34:88-93	Incorrect outcomes
Mumby C, Bernatavicius G, Zhang J, Myers J. Randomised controlled trial of metformin treatment versus standard diabetes antenatal care in women with mild fasting hyperglycaemia diagnosed in pregnancy: a pilot study BJOG. 2016;123:28-9	Incorrect study type
NCT02947503. Pregnancy Outcomes: effects of Metformin Study (POEM Study). ClinicalTrials.gov. 2016;0	Incorrect study type
NCT00812903. Randomized controlled, multicenter study evaluating treatment of glucose intolerance in pregnancy. ClinicalTrials.gov. 2008;0	Incorrect study type

3. Subgroup analysis of clinical outcomes based on pharmacological management

Figures 6-7 present subgroup analyses of the relative risk of emergency caesarean sections (C-sections) and neonatal hypoglycaemia, respectively, across different pharmacological treatments. 
